# Intracellular Ionic Strength Sensing Using NanoLuc

**DOI:** 10.3390/ijms22020677

**Published:** 2021-01-12

**Authors:** Tausif Altamash, Wesam Ahmed, Saad Rasool, Kabir H. Biswas

**Affiliations:** College of Health & Life Sciences, Hamad Bin Khalifa University, Education City, Qatar Foundation, Doha 34110, Qatar; taltamash@hbku.edu.qa (T.A.); wahmed@hbku.edu.qa (W.A.); srasool@hbku.edu.qa (S.R.)

**Keywords:** bioluminescence, biosensor, ionic strength, NanoLuc

## Abstract

Intracellular ionic strength regulates myriad cellular processes that are fundamental to cellular survival and proliferation, including protein activity, aggregation, phase separation, and cell volume. It could be altered by changes in the activity of cellular signaling pathways, such as those that impact the activity of membrane-localized ion channels or by alterations in the microenvironmental osmolarity. Therefore, there is a demand for the development of sensitive tools for real-time monitoring of intracellular ionic strength. Here, we developed a bioluminescence-based intracellular ionic strength sensing strategy using the Nano Luciferase (NanoLuc) protein that has gained tremendous utility due to its high, long-lived bioluminescence output and thermal stability. Biochemical experiments using a recombinantly purified protein showed that NanoLuc bioluminescence is dependent on the ionic strength of the reaction buffer for a wide range of ionic strength conditions. Importantly, the decrease in the NanoLuc activity observed at higher ionic strengths could be reversed by decreasing the ionic strength of the reaction, thus making it suitable for sensing intracellular ionic strength alterations. Finally, we used an mNeonGreen–NanoLuc fusion protein to successfully monitor ionic strength alterations in a ratiometric manner through independent fluorescence and bioluminescence measurements in cell lysates and live cells. We envisage that the biosensing strategy developed here for detecting alterations in intracellular ionic strength will be applicable in a wide range of experiments, including high throughput cellular signaling, ion channel functional genomics, and drug discovery.

## 1. Introduction

Ionic strength determined by the concentrations of different ionic species in a medium, both cations and anions, fundamentally influences electrostatic interactions in and among biomolecules and, therefore, regulates a plethora of cellular processes [1,2,3,4]. For instance, ionic strength affects the conformational stability and folding/unfolding behavior of highly charged proteins through charge screening, as detected by changes in fluorescence resonance energy transfer (FRET) efficiency [5] or a ligand-induced conformational change in a multidomain protein as determined from intramolecular bioluminescence resonance energy transfer (BRET) assays [6,7,8,9,10]. Furthermore, ionic strength is also known to affect enzymatic activity [11], aggregation and gel formation upon protein unfolding [12], the assembly of disease-causing amyloids [13,14], or the binding affinity and fluorescence intensity of amyloid-specific dye [15]. Similarly, the higher order assembly of the purified *Caenorhabditis elegans* LAF-1 protein formed by phase separation could be altered by changes in the concentration of sodium chloride in the solution [16]. Ionic strength also regulates the gene expression of osmolarity-sensitive genes, as detected by electron microprobe analysis and Northern blot assays of mRNA derived from various tonicity-sensitive genes [17,18], cellular volume by modulating the activity of ion channels [19], peptide–lipid bilayer interactions [20], and the diffusion of biomolecules on lipid membranes [21].

Variations in intracellular ionic strength can occur due to changes in the osmolarity of the cellular microenvironment, which may result in either osmotic swelling or shrinkage of cells. This can subsequently lead to the activation of regulatory volume decrease or increase mechanisms, respectively, through switching of volume-sensitive ion channels [22]. Intracellular signaling events, such as those leading to changes in the metabolic activity of the cell, can also affect intracellular ionic strength. For instance, arachidonic acid metabolites can activate certain potassium channels and inhibit voltage-sensitive chloride channels [22].

Given the above, developing sensitive tools for the sensing of intracellular ionic strength in live cells would be of significant value. Previous efforts in this direction include the incorporation of engineered, charged peptides [23] or sugar-binding proteins [24] into FRET sensors or membrane transporters in liposomes [25] or mitochondria [26]. However, fluorescence-based sensors are known to have the drawback of reduced dynamic range and low signal-to-noise ratio [27], in addition to inherent photobleaching and autofluorescence effects. Furthermore, the requirement for an external excitation source could potentially make them unsuitable for measuring ionic strength in photosensitive systems. Therefore, a simpler, more straightforward method will be preferred for ionic strength sensing.

Unlike fluorescence-based methods, bioluminescence systems have a broad dynamic range, high sensitivity, and operational simplicity [28,29,30,31]. We have previously found that the bioluminescence of Renilla luciferase (Rluc) is affected by the ionic strength [7], and hypothesized that this ionic strength effect can be extrapolated to other luciferases and used for ionic strength sensing. Rluc has a relatively short bioluminescence half-life and low thermal stability compared to Nano Luciferase (NanoLuc). NanoLuc was engineered from the 19 kDa subunit of the deep-see shrimp (*Oplophorus gracilirostris*) luciferease, and is relatively smaller in size compared to the firefly luciferase (Fluc) and Rluc, exhibits higher bioluminescence with a longer half-life, has higher physical and thermal stability and greater tolerance to pH, is uniformly expressed in cells without compartmentalization, and is not subjected to intracellular post-translational modifications [28]. Additionally, it utilizes a unique, highly specific substrate called furimazine in an ATP-independent fashion. Furimazine has minimal background bioluminescence compared to other luciferins [28], thus, exhibiting a higher signal-to-background ratio. All these properties of NanoLuc have led to its utility in a variety of applications including resonance energy transfer-based protein–protein interaction determination (BRET), gene regulation, and monitoring protein stability in diseased conditions [32,33,34,35,36,37,38,39]. In fact, a number of biosensing strategies have been devised based on the BRET phenomenon [40,41,42,43,44,45,46,47,48].

Here, we have explored the possibility of using NanoLuc for sensing ionic strength. Contrary to the increase in the bioluminescence of Rluc reported previously [7], increased buffer ionic strength negatively impacted the bioluminescence of a recombinantly purified NanoLuc protein. Furthermore, the reversibility of the effect alludes to the possibility of its use as an ionic strength sensor in live cells. Indeed, the NanoLuc bioluminescence could be altered by alterations in the extracellular ionic strength while fluorescence and BRET ratio of an mNeonGreen–NanoLuc fusion construct remained unchanged.

## 2. Materials and Methods

### 2.1. In Vitro Luciferase Assays

In vitro luciferase assays were performed using a recombinantly purified NanoLuc [49] (a kind gift from Dr. Nidhi Nath, Promega, Madison, WI, USA) in 1× Tris Buffered Saline (TBS) with 10% glycerol at a concentration of 10 nM (unless stated otherwise) and 10 µM furimazine (Promega, Madison, WI, USA) as a substrate (1000× stock prepared in ethanol diluted to 1× using 1× TBS). A sample without the NanoLuc protein was used as a control in the kinetic assay, while the concentration of the NanoLuc protein was varied between 10^−14^ to 10^−6^ M in the dose-dependent bioluminescence measurement assay. All bioluminescence measurements, unless stated otherwise, were acquired at 460 nm (peak emission wavelength of NanoLuc) [50].

In order to characterize the dependence of NanoLuc bioluminescence on the buffer ionic strength, the concentration of NaCl was varied from 55 to 4015 mM, and bioluminescence was measured with 1 s acquisition time at the wavelength of 460 nm after incubation with the indicated NaCl concentrations at 37 °C for 30 min. The bioluminescence activities of NanoLuc under varying NaCl concentrations were scanned between 400 and 650 nm wavelengths using a grating-based system with about 15 nm steps with each individual measurement acquired for 1 s.

The reversible effect of ionic strength on NanoLuc bioluminescence was determined by increasing and then subsequently decreasing NaCl concentration using buffered solutions at indicated time points during a continuous bioluminescence measurement with 1 s acquisition time. Briefly, the assay was initiated in a buffer containing 150 mM NaCl in a total volume of 50 μL. The assay was continued until 662 s, following which the instrument was paused and an equal volume of a Tris buffer containing 5 M NaCl was added to the assay to increase the total NaCl concentration to 2500 mM. Bioluminescence measurements were continued after the elevation of the buffer NaCl concentration until 1087 s, following which the instrument was paused and 200 μL of a Tris-buffer without NaCl was added to the assay in order to dilute the NaCl concentration by 3-fold (833 mM). Bioluminescence measurements were continued following the addition. All bioluminescence measurements were performed using Tecan SPARK^®^ multimode microplate reader (Tecan, Mannedorf, Switzerland).

### 2.2. In Silico Docking and Simulation

The effect of increased ionic strength on the bioluminescence of NanoLuc was analyzed by detecting and analyzing the catalytic site and its interaction with the substrate furimazine. This was performed by in silico docking of the substrate on NanoLuc using the AutoDock Vina algorithm and the available three-dimensional structure of NanoLuc (PDB: 5IBO; DOI: 10.2210/pdb5IBO/pdb). The structures of both NanoLuc (subunit A) and furimazine were prepared using AutoDockTools4 software (Molecular Graphics Laboratory Tools; http://mgltools.scripps.edu) [51]. Kollman charges and polar hydrogen atoms were added to both NanoLuc and furimazine and saved in the AutoDock (pdbqt) format. In silico docking was performed using the AutoDock Vina algorithm using a grid box with dimensions of 28 × 28 × 28 with 1 Å spacing and an exhaustiveness of 32. A total of 9 docking runs were performed, and the furimazine conformer showing the highest affinity (−8.0 kcal/mole) was used for further structural analysis.

Molecular dynamic simulation of NanoLuc under varying NaCl concentrations was set up using the QwikMD [52] plug-in available in Visual Molecular Dynamics (VMD) [53] and performed using NAMD2.12 [54]. Briefly, the protein was solvated using TIP3P water, charges were neutralized using NaCl, and the final NaCl concentrations were set to the indicated values. Simulations were run using the default parameters including a 2 fs time-step, a pressure of 1 bar, and a temperature of 300 K, controlled with a Langevin baro- and thermostat, respectively. The simulations under each NaCl concentration were run for a minimum of 100 ns, excluding the minimization, annealing and, equilibration steps. Simulation results were analyzed using tools available in VMD [53].

All structural analysis and figure preparations were performed using PyMOL (The PyMOL Molecular Graphics System, Version 2.0.0, Schrödinger, LLC; pymol.org; New York, NY, USA).

### 2.3. Cell Lysate and Live-Cell Assays

Cell lysate and live cell experiments were performed using human embryonic kidney (HEK) 293T cells (ATCC, Manassas, VA, USA) transfected with a plasmid construct expressing a fusion of the mNeonGreen and the NanoLuc protein (mNeonGreen–DEVD–NanoLuc) (Addgene: 98287; DEVD is the caspase 3 cleavage site) [55]. For assays with cell lysates, cells were washed in chilled Dulbecco’s phosphate-buffered saline (DPBS), harvested using a 2 mM ethylenediaminetetraacetic acid (EDTA) containing DPBS, and lysed after 48 h of transfection by sonication in a lysis buffer containing 50 mM HEPES (pH 7.5), 100 mM NaCl, 2 mM EDTA, 1 mM dithiothreitol (DTT), 1× protease inhibitor cocktail (ThermoFisher Scientific, Massachusetts, USA) and 10% glycerol [7,10]. Sonicated cell lysates were centrifuged at 4 °C for 1 h at 20,817 relative centrifugal force (RCF) and the supernatant was collected. NanoLuc bioluminescence, mNeonGreen fluorescence, and the bioluminescence resonance energy transfer (BRET) between NanoLuc (energy donor) and mNeonGreen (energy acceptor) was then determined using an appropriate volume of cell lysates for each data point after incubation with the indicated NaCl concentrations at 37 °C for 30 min using a Tecan SPARK^®^ multimode microplate reader. NanoLuc bioluminescence was determined at a wavelength of 460 nm and bioluminescence was acquired for 1 s immediately after addition of furimazine (1 in 200 dilution; Promega, Madison, WI, USA), whereas mNeonGreen fluorescence was determined by exciting the samples at 480 nm and emission was acquired at a wavelength of 520 nm. BRET between NanoLuc and mNeonGreen was determined by measuring bioluminescence at 460 nm and fluorescence at 520 nm after the addition of the furimazine substrate (1 in 200 dilution) and represented as a ratio of mNeonGreen fluorescence and NanoLuc bioluminescence.

Live cell experiments were performed in a similar manner, except that the cells were not lysed. Instead, they were harvested using a 2 mM EDTA-containing DPBS, which was exchanged by centrifugation with a buffer containing 50 mM HEPES, 150 mM NaCl, 5 mM KCl, 1 mM MgCl_2_, 11.1 mM D-glucose, 2 mM CaCl_2_, pH 7.4 [55,56,57,58]. The cells were incubated under the indicated NaCl concentrations 37 °C for 30 min, and all bioluminescence measurements were performed immediately after substrate addition. All experiments were performed in the same buffer and NanoLuc bioluminescence, mNeonGreen fluorescence, and BRET between NanoLuc and mNeonGreen was determined as indicated above for cell lysates using a Tecan SPARK^®^ multimode microplate reader.

### 2.4. Cell Culture and Transfection

Human embryonic kidney (HEK) 293T cells were maintained in Dulbecco’s modified Eagle’s media (DMEM) with 10% fetal calf serum (FCS), 120 mg/L penicillin and, 270 mg/L streptomycin at 37 °C in a humidified incubator with an atmosphere of 5% CO_2_. Cells were transfected using Lipofectamine transfection reagent (ThermoFisher Scientific, Waltham, MA, USA) following the manufacturer’s instructions.

### 2.5. Data Analysis and Figure Preparation

GraphPad Prism (version 8 for macOS, GraphPad Software, La Jolla, CA, USA; www.graphpad.com), in combination with Microsoft Excel, was used for data analysis and graph preparation. Figures were assembled using Adobe Illustrator.

## 3. Results and Discussion

In the current study, we have attempted to develop an ionic strength sensing strategy using the NanoLuc protein. This was premised on the observation of increased bioluminescence that we had reported for the Rluc protein in cell lysates previously [7]. Towards this, we characterized the bioluminescence of a recombinantly purified NanoLuc in vitro using the substrate furimazine (Supplementary Figure S1A). As has been reported previously [28,59], NanoLuc showed a sustained activity for about 30 min, while there was almost negligible bioluminescence from the blank sample that did not contain the enzyme (Supplementary Figure S1B). Furthermore, we determined the bioluminescence with a number of NanoLuc dilutions (Supplementary Figure S1C), which showed significantly higher activity compared to the control at a minimum concentration of 100 fM. However, to be able to adequately detect any alterations in NanoLuc bioluminescence under altered buffer ionic strengths, we selected a concentration of 10 nM for subsequent experiments, unless stated otherwise.

**Figure 1:**
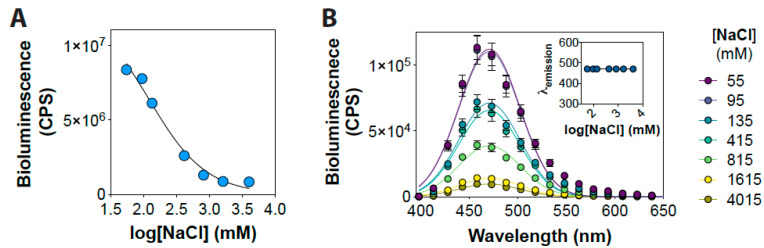
NaCl concentration-dependent NanoLuc activity. (**A**) Graph showing NanoLuc bioluminescence as a function of NaCl concentration. (**B**) Graph showing a wavelength scan of NanoLuc bioluminescence at indicated NaCl concentrations. Inset shows a graph emission max wavelength obtained from Gaussian fitting of the bioluminescence spectra. Data shown are mean ± standard error of mean (SEM) of 5 measurements from a representative experiment.

We investigated the effect of altered buffer ionic strength on the bioluminescence of NanoLuc in order to establish its utility in sensing ionic strength changes. For this, we incubated the NanoLuc protein with a range of NaCl concentrations (from 55 to 4015 mM) and measured the bioluminescence of the protein under these conditions. Incubation of the protein with increasing concentrations of NaCl resulted in a continuous decrease in its bioluminescence with an IC_50_ value of 121 ± 88 mM (from 1.1 ± 0.2 × 10^5^ CPS at 55 mM to 9.5 ± 0.3 × 10^3^ CPS at 4015 mM; ~22-fold decrease) (Figure 1A). However, no change in the spectral properties of the bioluminescence was observed (Figure 1B). While this is in contrast to the observations made with Rluc [7], a negative effect of increased buffer NaCl concentration has previously been reported for both NanoLuc and Fluc proteins [28]. Overall, these results indicate that the increase in the buffer NaCl concentration results in a decrease in the catalytic activity of the protein.

**Figure 2:**
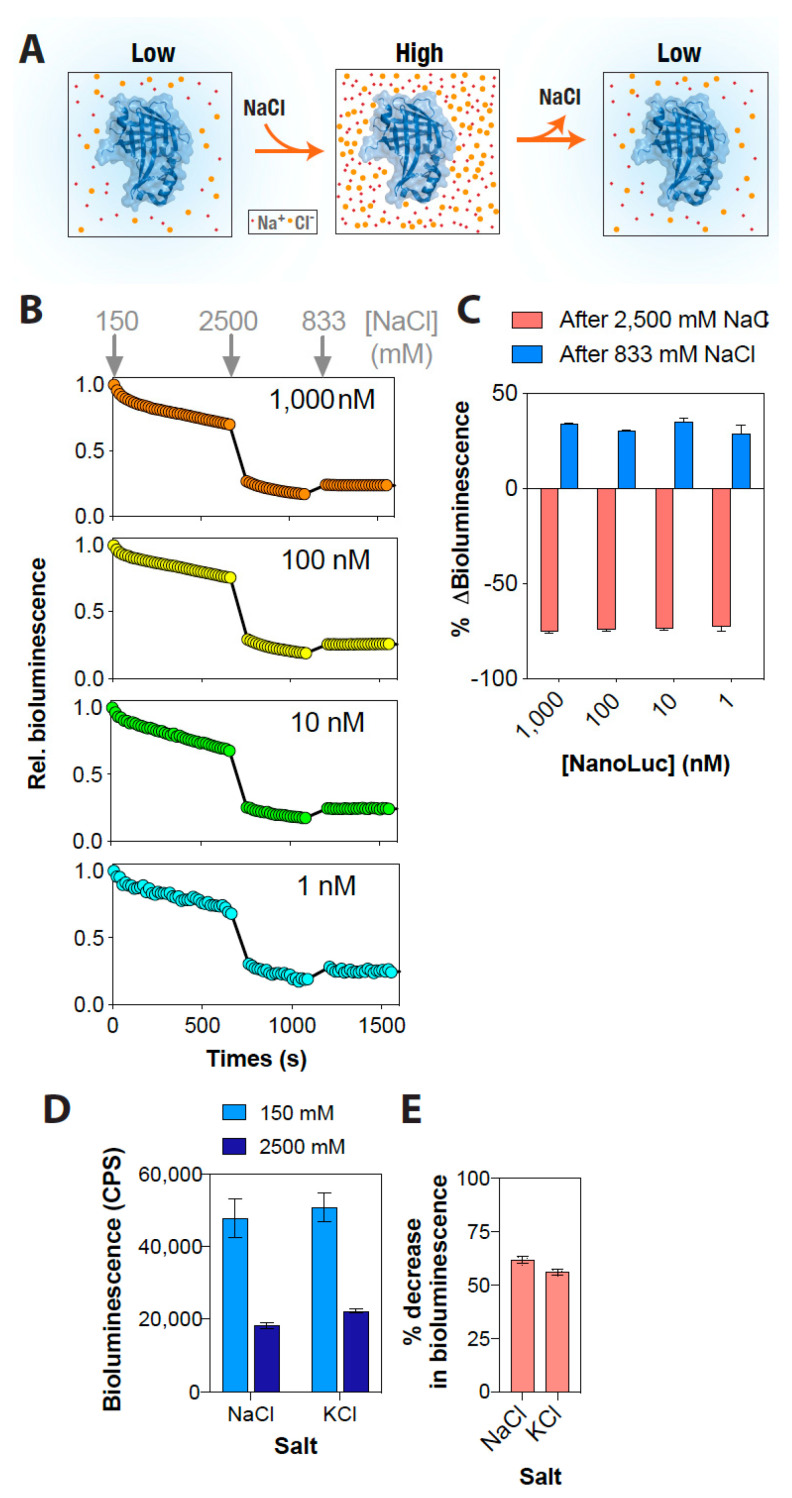
The effect of NaCl on NanoLuc activity is reversible. (**A**) Schematic representation showing the reversible effect of NaCl on NanoLuc bioluminescence activity. (**B**) Graph showing relative bioluminescence of indicated NanoLuc concentrations as a function of time at 150 mM NaCl, which was then increased to 2500 mM and then reversed back to 833 mM. (**C**) Graph showing the percentage change in NanoLuc bioluminescence with consecutive increases and decreases in NaCl concentration. (**D**) Graph comparing NanoLuc bioluminescence at the indicated concentrations of NaCl and KCl. (**E**) Graph showing percentage decrease in NanoLuc bioluminescence with increasing salt concentration as indicated in (**D**). Data shown are mean ± standard deviation (SD) of 5 measurements from a representative experiment.

Although a decrease in the NanoLuc bioluminescence with increasing NaCl concentration was promising, it was critical to test the reversibility of this effect for it to be used as a rapid and dynamic ionic strength sensor. For this, the bioluminescence of a range of NanoLuc concentrations were determined continuously, while the buffer NaCl concentration was increased from 150 mM to 2500 mM and then reverted to 833 mM (Figure 2A). This experiment revealed that the NanoLuc bioluminescence changed rapidly with an increase in the buffer NaCl concentration: increasing NaCl concentration from 150 mM to 2500 mM (16.7-fold) resulted in about a 75% decrease, while a subsequent three-fold dilution of the buffer NaCl concentration to 833 mM resulted in about a 30% increase in the activity across all NanoLuc concentrations tested (Figure 2B,C). We note that the incomplete increase in the NanoLuc bioluminescence at 833 mM NaCl concentration perhaps reflects a combination of an inhibitory effect of the still-higher NaCl concentration (833 vs. 150 mM) and substrate unavailability due to enzymatic oxidation (a continuous decay in the bioluminescence). These results indicate that the effect of buffer NaCl concentration on NanoLuc bioluminescence is reversible, thus suggesting its suitability for monitoring real-time alterations in ionic strength. More importantly, this reversible effect indicates that the decrease in activity observed as a result of an increase in the ionic strength is not due to changes in the protein structure, in agreement with results obtained from molecular dynamics simulations of the protein, which revealed no specific large scale structural changes in the protein under higher salt concentrations (Supplementary Figure S2). Instead, it likely reflects that this effect is a consequence of either electrostatic screening or an alteration in the solvation of the active site.

**Figure 3:**
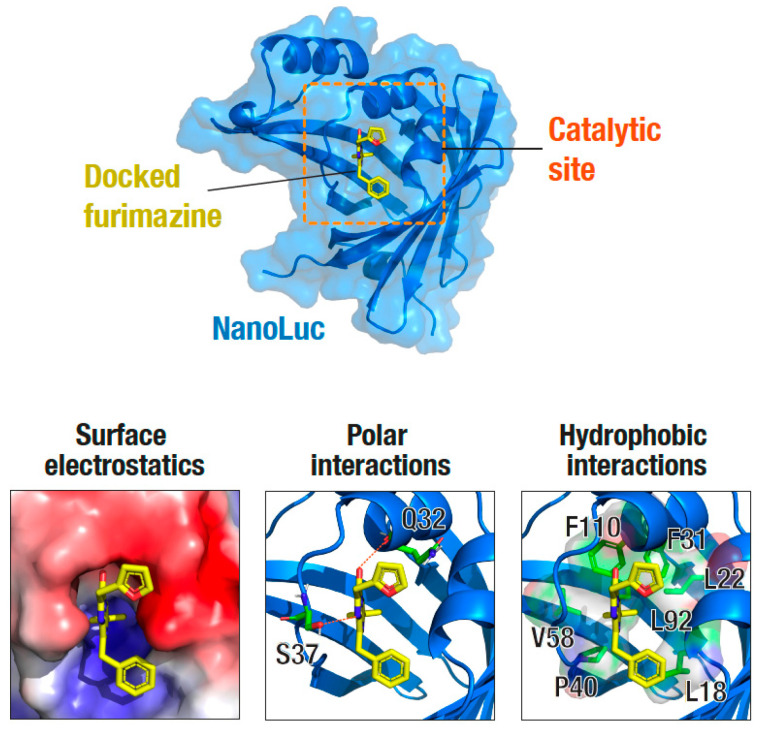
NanoLuc catalytic site structural analysis. Top panel: Surface and cartoon representation of the NanoLuc structure (PDB: 5IBO) showing the docked furimazine substrate at the catalytic site. The substrate, furimazine, was docked using the Autodock Vina software. Bottom panels: zoomed-in view of the catalytic site showing surface electrostatics (left), polar interactions (middle) and hydrophobic interactions (right) formed by furimazine.

The effect of increased buffer NaCl concentration on NanoLuc bioluminescence could be recapitulated with KCl, because similar decreases were observed with using it as well (Figure 2D,E). These results indicate that the effect of increased buffer ionic strength on the NanoLuc bioluminescence is generic. We then attempted to delineate the mechanism by which increased buffer ionic strength could potentially impact the bioluminescence output of NanoLuc. Unlike the case for Rluc [28], there are no structures of NanoLuc available with the substrate to enable a straightforward interpretation of the results. Therefore, we first computationally docked the substrate, furimazine, on the available structure of NanoLuc (PDB: 5IBO) using the AutoDock Vina algorithm, and then analyzed the interaction of the substrate with the protein (Figure 3). This revealed that the NanoLuc–furimazine interaction is driven by both polar (Q32 and S37) as well as hydrophobic interactions (L18, L22, F31, P40, V58, F110) (Figure 3), suggesting that the increased ionic strength likely screens the electrostatic interactions as well as disrupts the hydrophobic interactions leading to a decrease in the binding of the substrate to NanoLuc, and thus, a reduction in the bioluminescence. Such effects have been well documented in the literature, including substrate binding to the Na, K-ATPase [59], ligand binding to acetylcholinesterase enzyme [60], and quinacrine binding to the nicotinic acetylcholine receptor [61].

**Figure 4:**
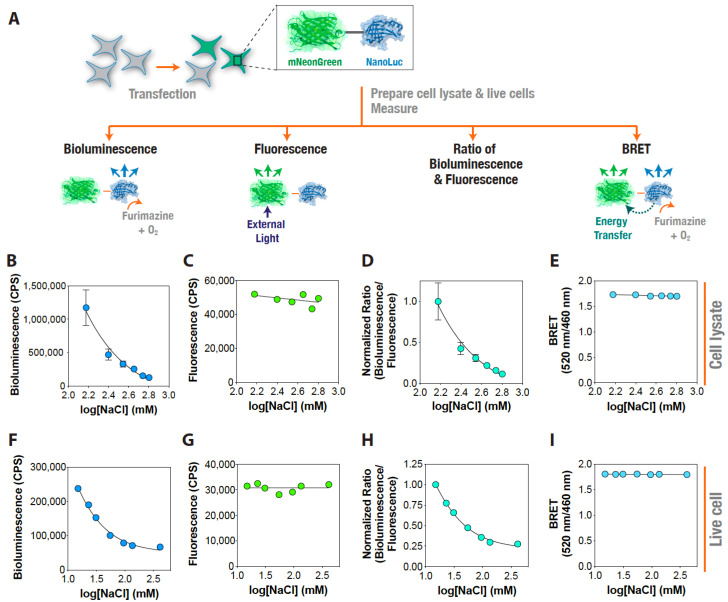
Ionic strength sensing using an mNeonGreen–NanoLuc fusion construct. (**A**) Schematic representation of the experiment showing transfection of cells with mNeonGreen–NanoLuc fusion construct, preparation of cell lysates and live cells, and bioluminescence, independent fluorescence, and BRET measurements. (**B**–**I**) Graphs showing bioluminescence: (**B**,**F**) independent mNeonGreen fluorescence; (**C**,**G**) normalized ratio of bioluminescence and mNeonGreen fluorescence (**D**,**H**); and BRET (ratio of 520 nm/460 nm emissions) (**E**,**I**); measured in cell lysates (**B**–**E**); and live cells (**F**–**I**) at the indicated NaCl concentrations. Data shown are mean ± standard deviation (SD) of 5 measurements from a representative experiment.

Having established the possibility of using the recombinantly purified NanoLuc protein as a dynamic reporter of ionic strength, we decided to test it in more complex samples, such as cell lysates and live cells. In order to control for undesired effects such as protein unfolding and changes in protein expression levels, we used a fusion construct containing mNeonGreen fluorescent protein along with the NanoLuc protein [55], and determined ratiometric changes in the bioluminescence normalized by the fluorescence output under different NaCl concentrations. HEK293T cells transfected with the plasmid were used to prepare cell lysates expressing the protein construct and incubated under the indicated NaCl concentrations with constant pH conditions (Figure 4A). Following incubation, we independently determined the mNeonGreen fluorescence (excitation at 480 nm and emission at 520 nm) and NanoLuc bioluminescence (emission at 460 nm). Additionally, we measured the BRET efficiency of the fusion construct under the same conditions by measuring light output at 520 nm (acceptor, mNeonGreen emission) and 460 nm (donor, NanoLuc emission) to rule out any Caspase 3 mediated cleavage of the fusion construct under the tested conditions. Lysates prepared from cells expressing the mNeonGreen–NanoLuc construct showed a decrease in the NanoLuc bioluminescence with increasing NaCl concentration in the buffer (Figure 4B), while the mNeonGreen independent fluorescence largely remained unaltered (Figure 4C). A ratiometric analysis of the NanoLuc bioluminescence and mNeonGreen independent fluorescence at each of the NaCl concentrations tested showed a NaCl concentration-dependent decrease in the ratio (Figure 4D), indicating that the ratio of NanoLuc bioluminescence and mNeonGreen independent fluorescence could be used as a reliable indicator of buffer ionic strength. Importantly, the BRET efficiency—which reflects the energy transfer from NanoLuc (donor) to mNeonGreen (acceptor) in the absence of any external source of light—of the fusion construct did not change under these conditions (Figure 4E) indicating that no undesired proteolysis or a conformational change of the fusion protein occurred in the cells under the experimental conditions. Similar results were obtained with live cells (Figure 4F–I), except that the decrease in NanoLuc bioluminescence plateaued after 135 mM external NaCl concentration. This perhaps suggests an underlying intracellular ionic strength regulatory mechanism in live cells by which they attempt to maintain an upper limit for ionic strength, which, if exceeded, may result in cellular malfunction and, eventually, in cell death. Together, these results indicate that NanoLuc bioluminescence could be utilized for monitoring ionic strength changes in both cell lysates as well as live cells (albeit up to a certain maximum ionic strength). Furthermore, this establishes a general strategy of combining independent fluorescence (in this case, mNeonGreen, but potentially any other fluorescence protein) and NanoLuc bioluminescence measurements using any such pairs of NanoLuc-based BRET constructs to ratiometrically determine changes in ionic strength, either in vitro or in live cells.

## 4. Conclusions

To conclude, we have shown that the bioluminescence of the recombinantly purified NanoLuc protein is regulated by the ionic strength of the buffer, and this phenomenon can be utilized for detecting alterations in the ionic strength changes in more complex samples such as cell lysates and live cells. It appears that an increase in the buffer ionic strength alters the interaction of the substrate with NanoLuc, leading to an effective decrease in the bioluminescence of the protein. We believe that the ratiometric ionic strength sensing strategy combining independent fluorescence and bioluminescence measurements developed here will find application in a variety of research areas, including ion channel functional genomics and drug discovery.

## Data Availability

The data presented in this study are available on request from the corresponding author.

## Supplementary Materials

The following are available online at https://www.mdpi.com/1422-0067/22/2/677/s1, Figure S1: In vitro NanoLuc characterization, Figure S2: Structural stability of NanoLuc monitored using molecular dynamics simulation, Figure S3: Effect of other salts on NanoLuc bioluminescence.
