# Supplementary material for: Intracellular Ionic Strength Sensing Using NanoLuc
Source: Int J Mol Sci. 2021 Jan 12;22(2):677. doi: 10.3390/ijms22020677 (PMC7826950; doi:10.3390/ijms22020677)
Supplement: Supplementary file 1 [file ijms-22-00677-s001.pdf]

Supporting information

Intracellular Ionic Strength Sensing Using NanoLuc

Tausif Altamash[1], Wesam Ahmed[1], Saad Rasool[1], Kabir H Biswas[1,*]

Affiliation:

[1]College of Health & Life Sciences, Hamad Bin Khalifa University, Doha, Qatar

*Correspondence: kbiswas@hbku.edu.qa

Supporting Figures

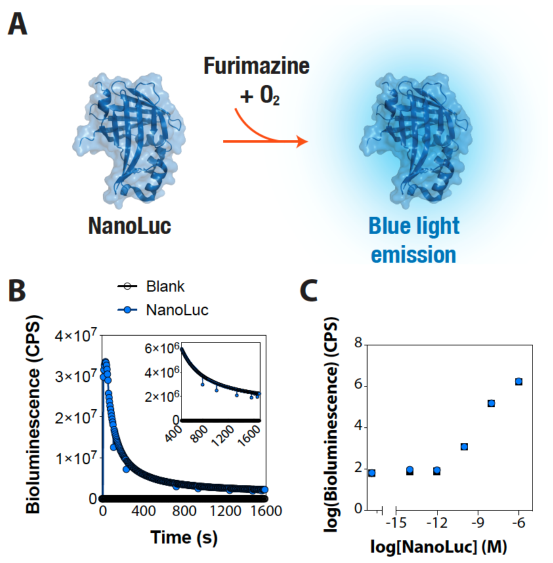

Supporting Figure 1. In vitro NanoLuc characterization. (A) Schematic representation of NanoLuc activity. (B) Graph showing luminescence (counts per second; CPS) of recombinantly purified NanoLuc as a function of time. Inset shows a sustained NanoLuc activity after 400 s. (C) Graph showing NanoLuc activity against its concentration. Data shown are mean ± standard deviation (SD) of 5 measurements from a representative experiment.

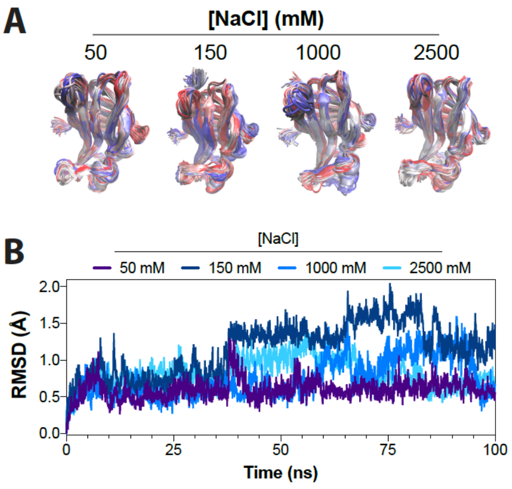

Supporting Figure 2. Structural stability of NanoLuc monitored using molecular dynamics simulation.

(A) Cartoon representation of NanoLuc structural evolution in a 100 ns all-atom, explicit solvent, molecular dynamics simulation at the indicated NaCl concentrations. Structures are color coded as per their trajectories. (B) Graph showing backbone (Ca) Root mean squared displacement (RMSD) values of NanoLuc structure over a 100 ns molecular dynamics simulation at the indicated NaCl concentrations. Data shown are from an individual experiment.
